# A novel R2R3-MYB transcription factor PpMYB5 assisting Ppbbx24-del positively regulates anthocyanin biosynthesis in ‘Red Zaosu’ pear

**DOI:** 10.1093/hr/uhaf300

**Published:** 2025-10-29

**Authors:** Shuran Li, Xiaofeng Liu, Fei Wang, Yanjie Zhang, Liyong Qi, Chunqing Ou, Shuling Jiang, He Li

**Affiliations:** Research Institute of Pomology, Chinese Academy of Agricultural Sciences, Key Laboratory of Horticultural Crops Germplasm Resources Utilization, Ministry of Agriculture and Rural Affairs, Xingcheng 125100, China; College of Horticulture, Shenyang Agricultural University, Shenyang 110866, China; Research Institute of Pomology, Chinese Academy of Agricultural Sciences, Key Laboratory of Horticultural Crops Germplasm Resources Utilization, Ministry of Agriculture and Rural Affairs, Xingcheng 125100, China; Research Institute of Pomology, Chinese Academy of Agricultural Sciences, Key Laboratory of Horticultural Crops Germplasm Resources Utilization, Ministry of Agriculture and Rural Affairs, Xingcheng 125100, China; Research Institute of Pomology, Chinese Academy of Agricultural Sciences, Key Laboratory of Horticultural Crops Germplasm Resources Utilization, Ministry of Agriculture and Rural Affairs, Xingcheng 125100, China; Research Institute of Pomology, Chinese Academy of Agricultural Sciences, Key Laboratory of Horticultural Crops Germplasm Resources Utilization, Ministry of Agriculture and Rural Affairs, Xingcheng 125100, China; Research Institute of Pomology, Chinese Academy of Agricultural Sciences, Key Laboratory of Horticultural Crops Germplasm Resources Utilization, Ministry of Agriculture and Rural Affairs, Xingcheng 125100, China; Research Institute of Pomology, Chinese Academy of Agricultural Sciences, Key Laboratory of Horticultural Crops Germplasm Resources Utilization, Ministry of Agriculture and Rural Affairs, Xingcheng 125100, China; College of Horticulture, Shenyang Agricultural University, Shenyang 110866, China

## Abstract

Anthocyanins are vital pigments that play a crucial role in the coloration of various fruits. Our previous study identified a mutant Ppbbx24-del protein in the ‘Red Zaosu’ pear that positively regulates anthocyanin biosynthesis. However, this mutant protein exhibited nucleo-cytoplasmic localization due to the lack of the NLS domain. We hypothesized that a transcription factor in ‘Red Zaosu’ pear interacts with Ppbbx24-del, facilitating its nuclear translocation for regulatory function. In this study, a PpMYB5 was screened by Y2H assay using the Ppbbx24-del as bait, which was an R2R3-MYB transcription factor and significantly up-expressed in ‘Red Zaosu’ compared to ‘Zaosu’. Pull-down, Y2H and BiFC assays confirmed that PpMYB5 could interact with both mutant Ppbbx24-del and common PpBBX24. Notably, co-expression experiments revealed that PpMYB5 facilitated the nuclear translocation of Ppbbx24-del. Transient expression assays in ‘Zaosu’ pear fruits demonstrated that PpMYB5 alone failed to induce anthocyanin accumulation, but its co-expression with Ppbbx24-del significantly enhanced the anthocyanin content of fruit peel compared to Ppbbx24-del alone. This synergistic effect was accompanied by significant upregulation of key anthocyanin biosynthetic genes, including *PpCHS* and *PpCHI*. Additionally, dual-luciferase assays demonstrated that PpMYB5 not only enhanced the activation effect on the promoters of *PpCHS* and *PpCHI* by Ppbbx24-del but also had the same effect on the promoter of *PpMYB5*. Our findings indicate that PpMYB5 and Ppbbx24-del form a crucial regulatory module that finely regulates anthocyanin synthesis in pear.

## Introduction

Anthocyanins are a type of polyphenolic compound that possess various beneficial properties, such as antioxidant, antibacterial, and anti-allergic effects [1]. A series of internal and external factors can impact anthocyanin production, including differences in gene expression and DNA sequences, temperature, light, soil type, humidity, and various environmental conditions [2]. Structural genes and regulatory genes are key determinants of anthocyanin accumulation and currently represent the primary focus of research [3, 4]. Structural genes directly affect the anthocyanin synthesis pathway by encoding essential enzymes such as phenylalanine ammonia-lyase (PAL), chalcone synthase (CHS), chalcone isomerase (CHI), flavanone3-hydroxylase (F3H), dihydroflavonol 4-reductase (DFR), anthocyanidin synthase (ANS), and flavonoid 3-O-glycosyl-transferase (UFGT) [5, 6]. Regulatory genes modulate structural gene expression, which in turn influences anthocyanin synthesis levels and compositional profiles [7, 8].

Research on regulatory genes is a significant area of interest, with the MYB being the largest family and regulating the expression of anthocyanin biosynthesis genes by forming MYB-bHLH-WD40 (MBW) protein complexes that combine with basic helix–loop–helix (bHLH) and W40 repeat proteins [9–13]. Plant MYB transcription factors are categorized into four subfamilies (1R-, R2R3-, R1R2R3-, and 4R-MYB) according to the number and configuration of MYB DNA-binding domains [14]. Studies demonstrated that R2R3-MYB is a crucial component involved in pigment synthesis in plants, significantly affecting the coloration of various fruits [15–17]. In apples, several MYB transcription factors, including MdMYB1 [18, 19], MdMYB3 [20], MdMYBA/10 [21], MdMYB11 [22], and MdMYB110a [23], are implicated in the synthesis of apple anthocyanins. In pears, the up-regulated expression of *PpMYB114* and *PpMYB10* leads to the accumulation of anthocyanin [24, 25]. PyMYB107 represses anthocyanin biosynthesis by competitively binding PybHLH3, thereby downregulating structural gene expression [26]. Previous studies indicated that MYB5, a member of the R2R3-MYB family, plays an essential role in the accumulation of anthocyanins in plants: Amato *et al*. [27] demonstrated that grape MYB transcription factors VvMYB5a and VvMYB5b, along with the WRKY factor VvWRKY26, collaboratively regulate anthocyanin accumulation through the formation of the MBW(W) (MYB-bHLH-WD40 with WRKY) complex; Shi *et al*. [28] found that ZjMYB5 activates the expression of both *ZjANS* and *ZjUGT79B1*, highlighting its crucial role in anthocyanin synthesis in jujube; In strawberry, FaMYB5 serves as an R2R3-MYB activator, playing a vital role in the MBW complex and actively regulating the biosynthesis of anthocyanins and proanthocyanidins [15]. Moreover, MYB5 may also contribute to anthocyanin synthesis in hawthorn [29]. The stable expression of *DoMYB5* in tobacco results in a significant increase in anthocyanin accumulation [30].

‘Red Zaosu’ (*Pyrus pyrifolia*, White Pear Group) originated as a bud mutation of its parent cultivar ‘Zaosu’ [8, 31, 32]. This cultivar features crisp flesh, flavorful juice, and a distinctive red peel, aligning with contemporary consumer preferences and conferring significant economic value [33–35]. We previously characterized a 14-bp deletion in PpBBX24’s coding region that distinguishes the red mutant ‘Red Zaosu’ from ‘Zaosu’ [31]. This deletion resulted in the formation of the mutant protein Ppbbx24-del, which significantly contributed to the coloration of ‘Red Zaosu’ and exhibited a function opposite to that of the common PpBBX24. Moreover, Ppbbx24-del lacked the nuclear localization signal (NLS) domain as a consequence of the deletion mutation. Transcription factors initiate gene expression by recognizing and attaching to specific DNA sequences within the nucleus [36]. Although Ppbbx24-del exhibited transcription factor-like activity, it lacked the NLS domain and co-localized in both the cytoplasm and nucleus. Therefore, we hypothesized that there may be a transcription factor in ‘Red Zaosu’ pear that interacts with Ppbbx24-del and facilitates its transport into the nucleus to execute its regulatory function.

In this study, we conducted yeast two-hybrid screening to identify proteins that interact with Ppbbx24-de, which revealed its association with the transcription factor PpMYB5. To investigate the nuclear translocation mechanism, we utilized subcellular localization tracking validating the role of PpMYB5 in promoting the nuclear import of Ppbbx24-del. We characterized their functions through transient expression and dual-luciferase assays to elucidate their synergistic effects on anthocyanin biosynthesis in ‘Red Zaosu’ pear. We further analyzed transcriptional regulation using EMSA and Y1H experiments, confirming distinct promoter activation patterns of *PpMYB5* by Ppbbx24-del and PpBBX24. Our investigation established a mechanistic model of cooperative anthocyanin regulation by Ppbbx24-del and PpMYB5, offering insights into plant anthocyanin biosynthesis and laying the groundwork for CRISPR-based gene editing to develop new red-skinned pear cultivars.

## Results

### PpMYB5 is a R2R3-MYB transcription factor

The pGBKT7 fusion plasmid, containing the full-length CDS of Ppbbx24-del, functioned as a bait vector in the yeast two-hybrid screening library. The yeast self-activation test results revealed a self-activation phenomenon in the pGBKT7-Ppbbx24-del + pGADT7 construct (Fig. S1A). At a concentration of 2.5 mM, 3-amino-1, 2, 4-triazole (3AT) effectively inhibited its self-activation phenomenon (Fig. S1B). Utilizing Ppbbx24-del cloned into the pGBKT7 bait vector, we conducted a yeast two-hybrid screen against a pear cDNA library. Sequential reporter gene assays, followed by DNA sequencing and BLAST homology analysis, identified candidate interacting proteins among the positive clones. The viability and interaction specificity of these clones were further validated using Yeast rotary validation (Fig. S2). The functions of these proteins were analyzed, resulting in the identification of the MYB protein PpMYB5, which was determined to be the most relevant to coloration. Consequently, PpMYB5 was subsequently validated as a key protein of interest.

**Figure 1:**
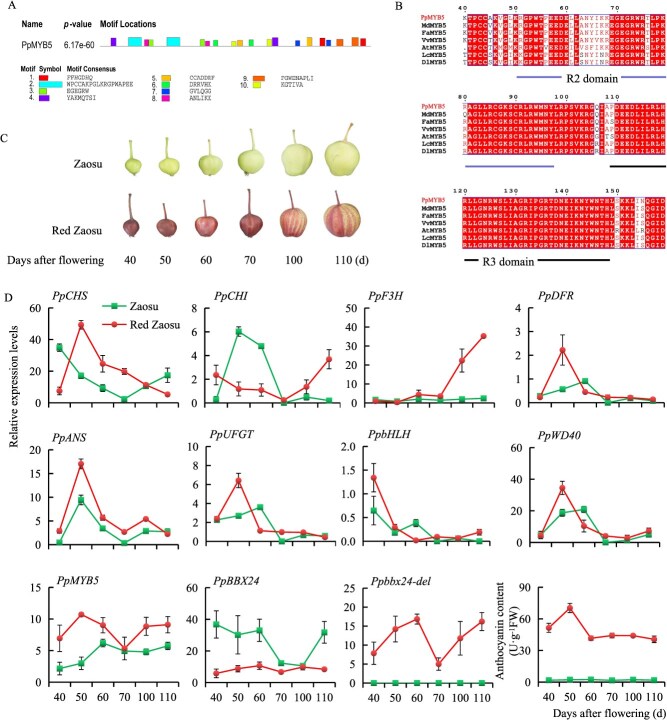
Structural characteristics and expression level analysis of anthocyanin related genes. (A) Functional domain analysis of PpMYB5. (B) R2R3-domains compared via ESPript 3.0. (C) Fruits of ‘Zaosu’ and ‘Red Zaosu’ at different developing stages. (D) The relative content of anthocyanin and the expression levels of related genes in ‘Zaosu’ and ‘Red Zaosu’ at different developing stages. The values were expressed as mean ± standard deviation of three independent biological replicates.

To investigate the potential role of PpMYB5 in regulating coloration in ‘Red Zaosu’, we initially analyzed its structural characteristics by amplifying the cDNA from both ‘Zaosu’ and ‘Red Zaosu’ pears using PCR. Cloning and sequencing revealed no differences in the *PpMYB5* sequence between the two varieties. The *PpMYB5* coding sequence was 1128 bp long and encoded 375 aa, which contained a MYB DNA-binding domain with two characteristic SANT domains (Fig. 1A). Phylogenetic tree analysis revealed that PpMYB5 is highly similar to the MYB5 proteins of *Arabidopsis thaliana* and several other fruit tree species (Fig. S3). ESPript analysis confirmed that these proteins possess conserved R2 and R3 domains and are classified as R2R3-type MYB transcription factors (Fig. 1B). We then measured the anthocyanin content and the expression levels of related structural genes in two pear cultivars across different developmental stages. Throughout fruit development, the anthocyanin content in the peel of ‘Red Zaosu’ remained consistently higher than that in ‘Zaosu’. Consistent with this, the expression level of PpMYB5 was also significantly elevated in ‘Red Zaosu’ (Fig. 1C and D).

### PpMYB5 interacted with Ppbbx24-del and PpBBX24

In the yeast two-hybrid screening library experiment, we found the protein PpMYB5 as a potential interactor of Ppbbx24-del. Considering the presence of both Ppbbx24-del and common PpBBX24 in the ‘Red Zaosu’ fruit, we predicted the three-dimensional structures of PpMYB5, Ppbbx24-del, and PpBBX24 using AlphaFold3, followed by docking analysis with HDOCK. PLIP analysis indicated that PpMYB5 and Ppbbx24-del interact strongly, as evidenced by 11 pairs of hydrogen bonds, one salt bridge, and numerous hydrophobic interactions (Fig. S4A). Additionally, PpMYB5 interacted with PpBBX24, as indicated by four pairs of hydrogen bonds, one salt bridge, and numerous hydrophobic interactions (Fig. S4B).

**Figure 2:**
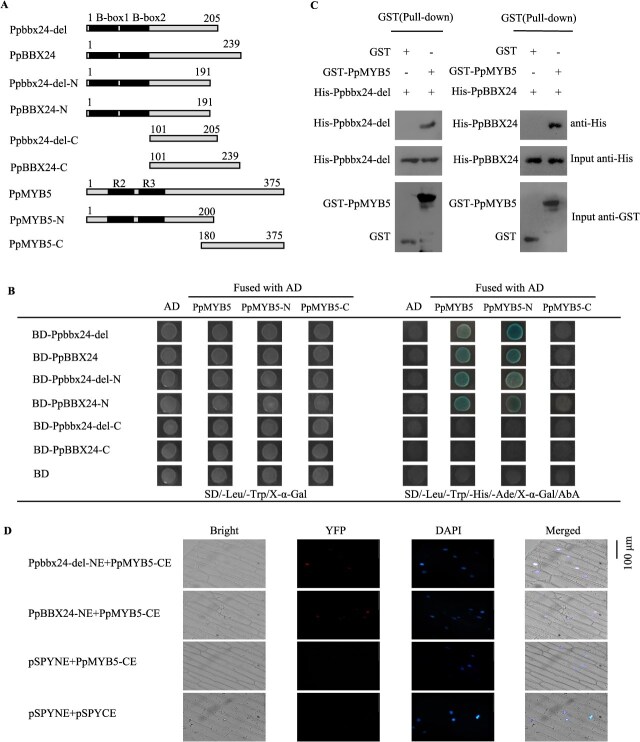
The interaction between PpMYB5 and Ppbbx24-del/PpBBX24. (A) Schematic diagram showing the different amino acid residues of Ppbbx24-del/PpBBX24 and PpMYB5 used in yeast two-hybrid assays. Ppbbx24-del/PpBBX24 and PpMYB5 are full-length proteins. Ppbbx24-del-N and PpBBX24-N are N-terminal parts of Ppbbx24-del and PpBBX24. Ppbbx24-del-C and PpBBX24-C are C-terminal parts of Ppbbx24-del and PpBBX24, respectively. PpMYB5-N and PpMYB5-C are N-and C-terminal parts of PpMYB5, respectively. (B) Y2H assays between PpMYB5 and both Ppbbx24-del and PpBBX24. (C) Pull-down assays of PpMYB5 and Ppbbx24-del/PpBBX24. Anti-His immunoblotting revealed successful pulldown of both Ppbbx24-del and PpBBX24 by GST-PpMYB5, but not by GST alone. Input controls demonstrated the loading of His-tagged proteins (anti-His) and GST-fusion proteins (anti-GST) before affinity purification. (D) BiFC assays between PpMYB5 and Ppbbx24-del/PpBBX24. Control combinations with empty pSPYNE and pSPYCE vectors.

We further confirmed the interaction between PpMYB5 and Ppbbx24-del/PpBBX24 by Y2H assay, pull-down assay, and BiFC experiment. In Y2H assays, the yeast strains co-transformed with PpMYB5 and Ppbbx24-del/PpBBX24 exhibited normal growth on selective media lacking four essential nutrients, thereby confirming the interaction between Ppbbx24-del/PpBBX24 and PpMYB5. Segmental experiments further confirmed that the R2R3 MYB domains of PpMYB5 interact with the B-box domains of Ppbbx24-del and PpBBX24 (Fig. 2A and B). In the pull-down assays, both His-Ppbbx24-del and His-PpBBX24 could be captured by GST-PpMYB5, but neither could be captured by the GST protein, suggesting a specific interaction between PpMYB5 and Ppbbx24-del/PpBBX24 (Fig. 2C). In the BiFC assays, the co-transformation of PpMYB5-CE and Ppbbx24-del-NE/PpBBX24-NE into onion epidermal cells led to the detection of yellow fluorescence in the nucleus, further substantiating the interaction between PpMYB5 and Ppbbx24-del/PpBBX24 (Fig. 2D).

### PpMYB5 assisted Ppbbx24-del translocation from the plasma membrane into the nucleus

**Figure 3:**
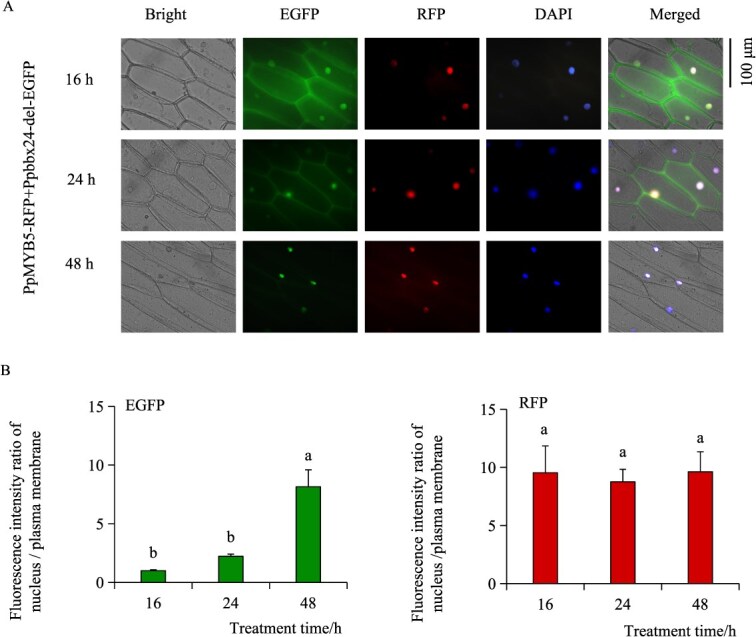
PpMYB5 is involved in the translocation process from the plasma membrane to the cell nucleus in Ppbbx24-del. (A) The subcellular transport of Ppbbx24-del and PpMYB5. (B) The ratio of fluorescent intensity in the nucleus compared to the plasma membrane during the nuclear entry process of Ppbbx24-del and PpMYB5. The values were expressed as mean ± standard deviation of three independent biological replicates. Different letters denote significant differences (*P* < 0.05; ANOVA, Tukey's test).

Our previous study indicated that Ppbbx24-del lacks the NLS domain, but it can still function as a transcription factor [31]. This suggests that additional transcription factors may assist in its entry into the nucleus. To investigate whether PpMYB5 assists in this process, we co-expressed PpMYB5-RFP and Ppbbx24-del-EGFP in onion epidermal cells using transient transfection. We found that co-expression of PpMYB5-RFP and Ppbbx24-del-EGFP led to initial co-localization of EGFP fluorescence in both the nucleus and cytoplasm, with more intense signals observed at the plasma membrane. Over time, the EGFP fluorescence signal exhibited a gradual decrease in co-localization at the plasma membrane and an increase in localization within the nucleus. The RFP signal remained consistently localized in the nucleus (Fig. 3A). The fluorescence intensity ratio of Ppbbx24-del in the nucleus increased progressively over time, whereas the fluorescence intensity ratio of RFP fluorescence signal of PpMYB5 in nucleus and cytoplasm did not change significantly (Fig. 3B). Additionally, we individually introduced Ppbbx24-del-EGFP and PpMYB5-RFP into onion epidermal cells to investigate the subcellular localization of PpMYB5 and Ppbbx24-del by continuously monitoring their fluorescent signals. The results showed that throughout the treatment period of Ppbbx24-del-EGFP transformation, the EGFP fluorescent signals showed consistent co-localization in both the nucleus and cytoplasm, without any observable nuclear import (Fig. S5A). In contrast, after the transformation of PpMYB5-RFP, the RFP fluorescent signals consistently localized within the nucleus throughout the observation period (Fig. S5B). Our findings indicate PpMYB5 is essential for Ppbbx24-del translocation from the plasma membrane to the nucleus.

### PpMYB5 assisted Ppbbx24-del in promoting anthocyanin accumulation

**Figure 4:**
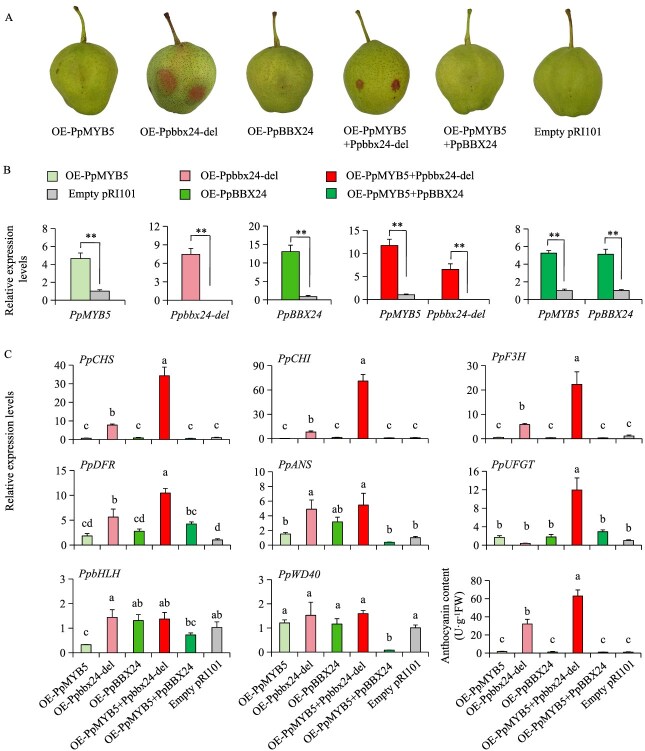
Effects of *PpMYB5*, *Ppbbx24-del,* and *PpBBX24* on pear anthocyanin accumulation. (A) Transient expression of *PpMYB5*, *Ppbbx24-del,* and *PpBBX24* in ‘Zaosu’. (B) Transcript levels of *PpMYB5*, *Ppbbx24-del,* and *PpBBX24* in the fruit peels of ‘Zaosu’. ^**^P < 0.01 (two-tailed Student’s *t*-test). (C) Transcript levels of anthocyanin structural genes and anthocyanin contents were measured in the fruit peels of ‘Zaosu’. The values were expressed as mean ± standard deviation of three independent biological replicates. Different letters denote significant differences (*P* < 0.05; ANOVA, Tukey’s test).

Our previous study demonstrated that Ppbbx24-del enhances fruit coloration in ‘Zaosu’ [31]. To determine whether PpMYB5 affects these results, we transiently transformed *PpMYB5* along with *Ppbbx24-del* and *PpBBX24* into ‘Zaosu’ pear (Fig. 4A). After transient expression, there were significant alterations in the expression levels of *PpMYB5*, *Ppbbx24-del*, and *PpBBX24* genes at the injection sites (Fig. 4B). Notably, overexpression of *Ppbbx24-del* (OE-*Ppbbx24-del*) and the co-overexpression of *PpMYB5* and *Ppbbx24-del* (OE-*PpMYB5* + *Ppbbx24-del*) led to anthocyanin accumulation at the injection sites. In contrast, individual overexpression of *PpMYB5* (OE-*PpMYB5*), *PpBBX24* (OE-*PpBBX24*), and co-injection of both *PpMYB5* and *PpBBX24* (OE-*PpMYB5* + *PpBBX24*), as well as injection of the empty vector (Empty pRI101), did not lead to significant anthocyanin accumulation. Quantitative analysis revealed synergistic effects between *PpMYB5* and *Ppbbx24-del*, with their co-expression inducing a 60-fold increase in peel anthocyanin content compared to controls, twice the accumulation level observed with Ppbbx24-del alone (30-fold increase). Neither *PpMYB5* nor *PpBBX24* single overexpression, nor their co-expression, significantly enhanced anthocyanin production (Fig. 4C). Furthermore, the combination of PpMYB5 and Ppbbx24-del most effectively activated the expression of structural genes, particularly resulting in a 30-fold upregulation of *PpCHS* and a 70-fold upregulation of *PpCHI* compared to the control. The activation levels significantly exceeded those from *Ppbbx24-del* single overexpression, with increases of 7- to 8-fold for both genes. In contrast, other combinations exhibited minimal effects (Fig. 4C). These results strongly indicate that the functional interaction between *PpMYB5* and *Ppbbx24-del* potently enhances pear anthocyanin biosynthesis.

We further validated the function of *PpMYB5* in pear pigmentation by its stable transformation in pear calli. Following light induction, no detectable anthocyanin accumulation was observed in OE-*PpMYB5* calli (Fig. S6A and B). RT-qPCR analysis of anthocyanin biosynthetic genes indicated no significant differences in transcript levels between OE-*PpMYB5* and wild-type calli (Fig. S6C). Collectively, the results demonstrate that the expression of *PpMYB5* alone does not significantly impact anthocyanin biosynthesis.

**Figure 5:**
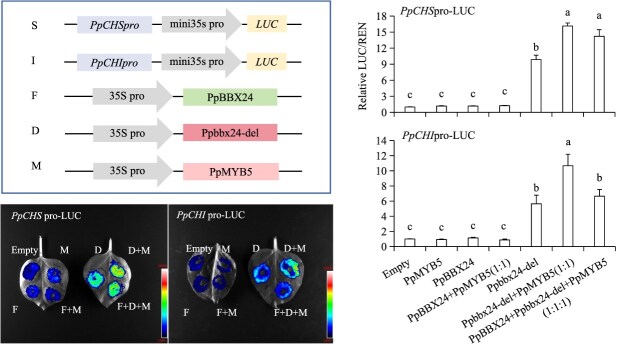
Dual luciferase assay confirms the effects of PpMYB5, Ppbbx24-del, and PpBBX24 on the activities of *PpCHS* and *PpCHI* promoters. The concentration of *A. tumefaciens* was consistently maintained across all co-injection treatments. The values were expressed as mean ± standard deviation of three independent biological replicates. Different letters denote significant differences (*P* < 0.05; ANOVA, Tukey’s test).

To investigate interactions between PpMYB5, Ppbbx24-del/PpBBX24 and the *PpCHS*/*PpCHI* promoters, we performed dual luciferase reporter assays. Expression of PpMYB5 alone did not affect the promoters’ activities of *PpCHS* and *PpCHI*. Notably, co-expression of PpMYB5 and Ppbbx24-del led to increased transactivation of the *PpCHS* and *PpCHI* promoters relative to Ppbbx24-del alone. In contrast, co-expression of PpMYB5 and PpBBX24 did not produce a significant effect on these promoters, akin to the effect of PpBBX24 alone. Co-transformation of PpMYB5, Ppbbx24-del, and PpBBX24 also enhanced promoter activation (Fig. 5). These findings indicate that PpMYB5 and Ppbbx24-del establish a regulatory complex that activates the transcription of anthocyanin structural genes.

### Ppbbx24-del promoted the expression of *PpMYB5* by binding to its promoter

**Figure 6:**
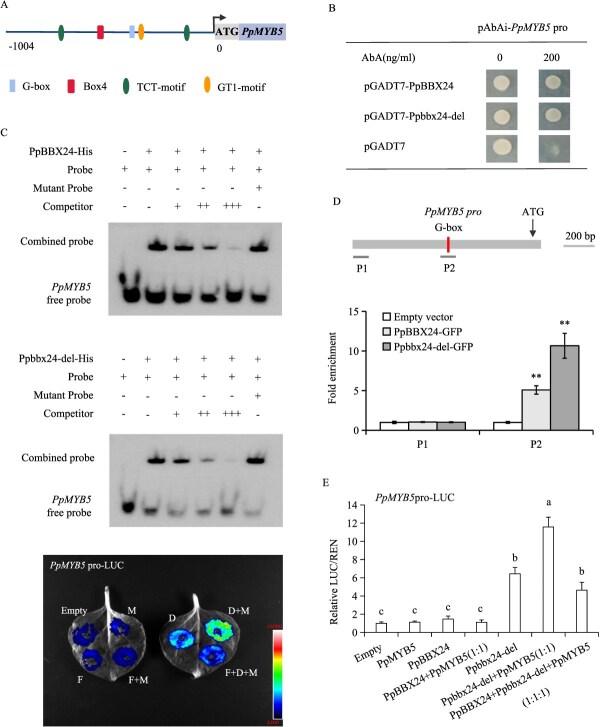
Binding of PpBBX24/Ppbbx24-del to *PpMYB5*. (A) Distribution of cis-elements in *PpMYB5* promoter. (B) Y1H assays between PpBBX24/Ppbbx24-del and the *PpMYB5* promoter. (C) The EMSA results demonstrate that PpBBX24/Ppbbx24-del specifically bind to the G-box motif located in the *PpMYB5* promoter. This experiment utilized unlabeled probes were used to perform competitive analysis. The symbols ‘−’ and ‘+’ denoted the absence and presence of the probes, respectively. (D) ChIP-qPCR analysis of PpBBX24 and Ppbbx24-del binding to the *PpMYB5* promoter. Chromatin from PpBBX24/Ppbbx24-del-GFP-overexpressing pear calli (*n* = 3 biological replicates) was cross-linked, immunoprecipitated with GFP antibody, and eluted DNA was analyzed by qPCR. Regions P1 and P2 were examined. Pear calli overexpressing GFP served as the negative control. ^**^P < 0.01 (two-tailed Student’s *t*-test). (E) Dual luciferase assays evaluate the effects of PpBBX24 and Ppbbx24-del on the activity of the *PpMYB5* promoter. The values were expressed as mean ± standard deviation of three independent biological replicates. Different letters denote significant differences (*P* < 0.05; ANOVA, Tukey’s test).

To explore the factors contributing to the elevated expression of *PpMYB5* in ‘Red Zaosu’, we cloned its promoter region. Analysis of the *PpMYB5* promoter showed identical sequences in ‘Red Zaosu’ and ‘Zaosu’, including a G-box cis-regulatory motif (Fig. 6A). In previous experiments, we demonstrated that both PpBBX24 and Ppbbx24-del bind to the G-box motif [31]. Subsequently, the binding was further verified using Y1H, EMSA and ChIP assays. The yeast cells containing the PpBBX24/Ppbbx24-del and *PpMYB5* promoters exhibited robust growth in the Y1H experiment (Fig. 6B). In the EMSA assays, both PpBBX24 and Ppbbx24-del bound to probes containing G-box elements but did not bind to the mutant probes (Fig. 6C). In the ChIP assays, PpBBX24 and Ppbbx24-del significantly enriched the G-box-containing P2 region of the *PpMYB5* promoter, indicating their binding (Fig. 6D). These findings suggest that both PpBBX24 and Ppbbx24-del interact with the promoter region of *PpMYB5*. Additionally, we assessed the activation effects of PpBBX24 and Ppbbx24-del on the promoter of *PpMYB5* using dual luciferase assays. Co-expression of PpMYB5 and Ppbbx24-del resulted in enhanced transactivation of the *PpMYB5* promoter compared to Ppbbx24-del alone. In contrast, co-expression of PpMYB5 and PpBBX24 did not produce a significant effect on the promoter, similar to the effects observed with PpMYB5 or PpBBX24 alone. The co-transformation of PpMYB5, Ppbbx24-del, and PpBBX24 enhanced promoter activity (Fig. 6E). These findings indicate that PpMYB5 and Ppbbx24-del interact to form a regulatory complex that enhances *PpMYB5* transcription.

## Discussion

### PpMYB5 assisted Ppbbx24-del translocation from the plasma membrane to the nucleus

Our prior work showed that ‘Red Zaosu’ exhibits about 50% replacement of *PpBBX24* with *Ppbbx24-del* compared to ‘Zaosu’ [31]. Ppbbx24-del lacks a 14-bp nucleotide sequence, which results in a modified C-terminal protein sequence that does not contain an NLS domain compared to PpBBX24 [33]. Subcellular localization analysis revealed that Ppbbx24-del exhibited nuclear-cytoplasmic co-localization. Furthermore, Ppbbx24-del can directly activate the expression of *PpCHS*, and *PpCHI*, thus significantly enhancing the synthesis and accumulation of anthocyanins [31]. This finding indicates that Ppbbx24-del functions as a transcription factor. Because transcription factors usually need to bind to nuclear DNA to function, we hypothesized that specific proteins assist with the nuclear entry of Ppbbx24-del. In this study, we identified PpMYB5 as an interacting partner of Ppbbx24-del through yeast two-hybrid screening (Fig. 1). BiFC results showed the interaction between Ppbbx24-del and PpMYB5 predominantly occurred in the nucleus (Fig. 2). We discovered that Ppbbx24-del initially localized to the plasma membrane and subsequently translocated to the nucleus following its interaction with PpMYB5 (Fig. 3). This mechanism parallels findings from studies demonstrating that plasma membrane-associated proteins can translocate into the nucleus in response to signaling cues [37, 38]. Although mitogen-activated protein kinases (MAPKs) typically reside in the cytoplasm and/or nucleus, they can translocate to the nucleus in a signal-dependent manner [39]. Similarly, bHLH039 relocates to the nucleus in the presence of FIT [40], while its interaction with the E3 ubiquitin ligase OsPIE3 alters PID2 localization, promoting nuclear complex formation [36]. Our results further indicated that interaction between Ppbbx24-del and the transcription factor PpMYB5 with nuclear localization capability altered the subcellular distribution of Ppbbx24-del, thereby facilitating the assembly of nuclear import complexes. Sequence analysis revealed that PpMYB5 contains two NLS domains (Fig. S7). Given that translation occurs on ribosomes in the cytoplasm, we hypothesize that the PpMYB5 protein is synthesized by ribosomes and subsequently interacts with Ppbbx24-del in the cytoplasm. This interaction facilitates the formation of a complex that contains a shared NLS. The NLS promotes the recognition of the complex by importin proteins, which then mediate its active transport into the nucleus through the nuclear pore complex. However, the exact molecular mechanisms underlying the PpMYB5-assisted nuclear trafficking of Ppbbx24-del necessitate further elucidation.

### The interaction of PpMYB5 with Ppbbx24-del promoted anthocyanin biosynthesis

Recent studies indicate that *PbMYB5* significantly enhances anthocyanin accumulation in pears [41]. Two *MYB5* homologs were identified in the reference genome of the ‘Zhongai 1’ pear cultivar. Sequence alignment revealed that the PpMYB5 protein sequence shares 100% identity with Pdr11g025100, while PbMYB5 exhibited higher similarity to Pdr3g017680 (Fig. S8). Moreover, significant differences were noted in both nucleotide and amino acid sequences between PpMYB5 and PbMYB5 (Figs S8 and S9). Our study demonstrated that the overexpression of *PpMYB5* alone does not significantly promote anthocyanin biosynthesis in pears. The transient expression of *Ppbbx24-del* led to a higher relative anthocyanin content at the injection site compared to the control group and significantly upregulated the structural genes *PpCHS* and *PpCHI*. In contrast, although PpMYB5 did not directly activate the *PpCHS* and *PpCHI* promoters, co-expressing the Ppbbx24-del-PpMYB5 complex further enhanced pear anthocyanin accumulation. Meanwhile, compared to *Ppbbx24-del* single expression, co-expression with *PpMYB5* significantly enhanced *PpCHS* and *PpCHI* transcript levels (Fig. 4). Dual-luciferase assays further confirmed that PpMYB5 assisted Ppbbx24-del in activating the expression of downstream genes (Fig. 5). Although the interactive relationship between PpMYB5 and PpBBX24 has been confirmed, the overexpression of the *PpBBX24*-*PpMYB5* complex did not significantly influence anthocyanin accumulation in ‘Zaosu’ (Figs 4 and 5). In the presence of Ppbbx24-del, PpBBX24, and PpMYB5, PpBBX24 competes with Ppbbx24-del for binding alongside PpMYB5. The activation of *PpCHI* observed following the injection of these three bacterial solutions in dual-luciferase assays was significantly weaker than that resulting from the injection of only the Ppbbx24-del and PpMYB5 complex (Fig. 5).

In this study, we found that the *PpMYB5* sequence is identical in both ‘Zaosu’ and ‘Red Zaosu’. But the expression level in ‘Red Zaosu’ is significantly higher than that in ‘Zaosu’ (Fig. 1). This finding suggests differences in transcriptional regulation between the two cultivars. Research showed that BBX proteins can bind to *MYB* gene promoters and modulate their transcription. In pears, PpBBX18 activates the expression of *PpMYB10* [32], while in lilies, LvBBX24 directly binds the G-box in the *LvMYB5* promoter, activating its transcription [42]. We cloned the *PpMYB5* promoter and validated its interaction with both Ppbbx24-del and PpBBX24 using EMSA, Y1H and ChIP assays. Notably, Ppbbx24-del exhibits significantly stronger activation than PpBBX24 (Fig. 6). This finding likely accounts for the elevated expression of *PpMYB5* in ‘Red Zaosu’.

**Figure 7:**
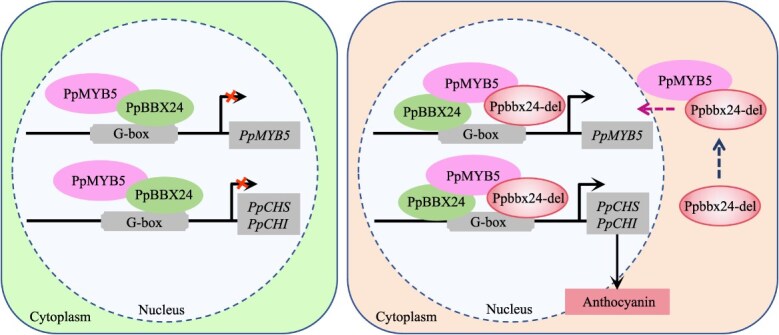
The synergistic regulation of anthocyanin biosynthesis by PpMYB5, PpBBX24, and Ppbbx24-del in ‘Zaosu’ (left) and ‘Red Zaosu’ (right).

In this study, the BBX protein PpMYB5, which plays a significant role in anthocyanin biosynthesis, functions as a cofactor for both Ppbbx24-del and PpBBX24. In ‘Zaosu’, neither PpBBX24 nor PpMYB5 can independently activate the promoters of their respective target genes. Conversely, in ‘Red Zaosu’, PpMYB5 interacts with the transcription factor Ppbbx24-del, promoting its translocation to the nucleus. Ppbbx24-del and PpMYB5 form a heterodimer complex that transcriptionally activates *PpMYB5*, *PpCHS*, and *PpCHI*. In contrast, the interaction between PpBBX24 and PpMYB5 impedes the formation of the functional complex involving Ppbbx24-del and PpMYB5, thus adversely affecting anthocyanin biosynthesis. In conclusion, the activation of anthocyanin structural gene expression by both Ppbbx24-del and PpMYB5 significantly exceeded the inhibitory effect of PpBBX24 on anthocyanin biosynthesis, leading to the red coloration observed in the peel of ‘Red Zaosu’ (Fig. 7). We identify new regulatory nodes within the anthocyanin transcriptional network and associated biosynthetic routes, highlighting the interaction between the complexes of Ppbbx24-del and PpMYb5 in *Rosaceae* fruit crops.

 The fruit peel colors of ‘Zaosu’ and ‘Red Zaosu’ are green and red, respectively. These color variations result from the functional differences among PpMYB5, PpBBX24, and the Ppbbx24-del complex. These differences lead to variations in the intensity of transcriptional regulation of target genes, particularly *PpCHS* and *PpCHI*. In ‘Zaosu’, PpBBX24 interacts with PpMYB5 without significantly affecting the expression levels of *PpCHS* and *PpCHI*. This interaction leads to minimal anthocyanin synthesis and sustains green cell integrity. In ‘Red Zaosu’, the transcription factor PpMYB5 promotes the translocation of Ppbbx24-del into the cell nucleus. The transcriptional activator Ppbbx24-del interacts with PpMYB5 to form a complex that strongly activates the expression of the downstream genes, including *PpCHS* and *PpCHI*. Furthermore, Ppbbx24-del and PpMYB5 significantly enhance the promoter activity of *PpMYB5*, leading to an increased abundance of *PpMYB5* in ‘Red Zaosu’. As a result, the transcript levels of *PpCHS* and *PpCHI* are significantly elevated, which promotes anthocyanin synthesis and imparts a red color to the fruit peel.

## Materials and methods

### Plant materials

The samples originated from Xingcheng, China (120°44′38″E, 40°37′9″N) at the Research Institute of Pomology (CAAS) orchard., where standard fertilization and irrigation methods were applied [31]. Tobacco plants (*Nicotiana benthamiana*), grown under standard conditions, were employed for transient expression.

### Yeast two-hybrid screening library

Both the library and the yeast two-hybrid screening library were constructed by ProNet Biotech Co., Ltd. A yeast two-hybrid library derived from pear was screened using the *Ppbbx24-del* constructed on the pGBKT7 vector as bait. Positive clones were analyzed using various reporter gene assays, DNA sequencing, and BLAST comparisons to identify proteins that interact with pGBKT7-Ppbbx24-del.

### Gene clone and expression analysis

Total RNA extraction, cDNA synthesis, and quantitative real-time PCR (qRT-PCR) followed established protocols [33]. Phylogenetic analysis was performed using MEGA 5.0. MYB domains were characterized with Pfam (pfam.xfam.org) and ESPript 3.0. Conserved domains and functional units were identified using NCBI CD-Search (ncbi.nlm.nih.gov). Quantitative PCR (RT-qPCR) was conducted with Luna Universal qPCR Master Mix (New England Biolabs, M3003X) using a Bio-Rad CFX96 Touch instrument [31]. RT-qPCR primers are detailed in Table S1.

### Molecular docking assay

AlphaFold3 was employed to predict the three-dimensional structures of PpMYB5, Ppbbx24-del, and PpBBX24. The structure exhibiting the highest pLDDT value was designated as the receptor protein and ligand protein. Protein–protein docking was performed using the HDOCKlite v1.1 local server [43, 44]. A detailed and systematic examination of the binding interfaces in protein–protein complexes was performed using the PLIP interaction analysis platform [45]. Subsequently, pyMOL was employed to enhance the understanding of the interactions.

### Pull-down assays

Constructs for *Ppbbx24-del* and *PpBBX24* were made in the pET-N-His-TEV vector (His tag), while the *PpMYB5* construct was made in the pET-N-GST-precision vector (GST tag). The resulting fusion proteins (His-PpBBX24, His-Ppbbx24-del, GST-PpMYB5) were expressed in *E. coli* BL21(DE3) and purified using a denatured His-tag purification kit (P2229S; Beyotime) for the His-tagged proteins and a GST-tagged protein purification kit (P2262; Beyotime) for GST-PpMYB5. His-PpBBX24 or His-Ppbbx24-del was individually incubated with GST-PpMYB5 using GST pull-down resin. Bound proteins were detected by Western blotting using an anti-His antibody.

### Y2H assays

The Y2H experiment refers to the method proposed by Li *et al.* [31]. The CDS of *PpBBX24*, *Ppbbx24-del*, and *PpMY5* were inserted into the pGBKT7 and pGADT7 vectors. Subsequently, the vectors were transformed into Y2HGold yeast cells utilizing the polyethylene glycol/lithium acetate method. Yeast was cultured on both two-deficient and four-deficient media, allowing for the observation of interactions by colony growth and color change.

### BiFC assays

The CDS of PpBBX24, Ppbbx24-del, and PpMYB5, excluding their termination codons, were cloned into pSPYNE and pSPYCE vectors. Subsequently, all combinations of *Agrobacterium tumefaciens GV3101* carrying these constructs, along with PpBBX24-NE/Ppbbx24-del-NE and PpMYB5-CE fusion proteins, were transiently co-expressed in onion epidermal cells [46]. Employing an Olympus IX51 fluorescence microscope coupled to a DP22 image acquisition system (Tokyo, Japan) to assess protein subcellular localization.

### Subcellular localization analysis

The CDS of *Ppbbx24-del* and *PpMYB5* were cloned into the pRI101 vector to produce EGFP- and RFP-tagged fusion proteins. *A. tumefaciens* GV3101, containing these constructs, was introduced into onion and incubated for 30 minutes. Following a dark incubation period on MS medium, fluorescence was observed at 16, 24, and 48 hours. The fluorescence intensity ratio between the cell nucleus and the membrane was measured using Image J software across various treatment durations.

### Transient expression in pear fruit

Pear fruits were infiltrated with *A. tumefaciens* GV3101 containing the recombinant vectors pRI101-*PpBBX24*, pRI101-*Ppbbx24-del* and pRI101-*PpMYB5*. After infiltration, the fruits were incubated in the dark for a minimum of 2 days, followed by 5 days of light exposure before phenotypic documentation. For RT-qPCR and anthocyanin content detection, peels surrounding the infiltration sites were collected [31].

### Transformation of pear calli

The pear calli were transformed using the same vector as described in Section Transient expression in pear fruit. Following a 3-day co-culture period, the calli were screened on cefotaxime (250 mg·l^−1^) and kanamycin (20 mg·l^−1^) supplemented MS solid medium under dark conditions; they were then subcultured biweekly. The calli were subjected to light treatment and subsequently observed after a 2-week period.

### Measurement of anthocyanin contents

The samples (1 g fresh weight) were extracted using 10 ml of 1% HCl/CH_3_OH at 4°C for about 24 hours in the dark. After extraction, the anthocyanin-rich supernatant was analyzed using UV–Vis spectrophotometry, with measurements taken at dual wavelengths of 553 nm and 600 nm. The relative anthocyanin concentration was quantified, with each 0.01 absorbance unit corresponding to one unit of anthocyanin content, following the standardized protocol [47].

### Dual-luciferase assays

This study builds on method of Zang *et al*. [48] with minor enhancements. The pGreenII800-LUC vector was ligated with the mini35S promoter to produce the mini35S-LUC recombinant construct. The promoter was connected with the above vector. Following rapid injection, the Tanon-5200 Multi Chemiluminescent Imaging System was utilized for imaging. Promoter activity was evaluated by quantifying fluorescence using a SPECTRA MAX 190 microplate reader and the Dual Luciferase Reporter Gene Assay Kit (RG027; Beyotime), calculating the LUC/REN activity ratio.

### EMSA assays

The G-box-containing probes are detailed in Table S2. Biotin labeling was performed with Beyotime’s EMSA Probe Labeling Kit (GS008; Shanghai, China). EMSA utilized Beyotime’s Chemiluminescent Kit (GS009) according to manufacturer protocols, and imaging was performed on a Bio-Rad ChemiDoc MP Imager to visualize results [49].

### Y1H assays

The CDSs were cloned into the pGADT7 vector, while the promoter fragment was ligated into the pAbAi vector. After co-transformation into Y1HGold yeast, binding activity was assessed by culturing transformants on SD/−Leu/AbA selection plates; growth confirms transcription factor-promoter interaction [50].

### Chromatin immunoprecipitation (ChIP)-qPCR analysis

The PpBBX24 and Ppbbx24-del CDSs were cloned into pRI101-GFP vectors and transformed into *A. tumefaciens* EHA105, respectively. Pear fruit calli were infected as described above. Preliminary calli treatment for ChIP-PCR followed Qi *et al*. [51]. ChIP assays were performed using the SimpleChip Plus Sonication Chromatin IP Kit according to the manufacturer’s instructions (catalog no. 56383; Cell Signaling Technology, Danvers, MA, USA). Chromatin was fragmented using a SCIENTZ-IID tor (SCIENTZ, NingBo, China) (1 second on, 1 second off, 120 cycles). Immunoprecipitation was performed using a GFP antibody (1 mg ml^−1^, catalog no. HT801–01; Transgen). Enriched chromatin was analyzed by qPCR. Fruit calli were independently infected three times to generate lines; each line underwent one ChIP analysis. Enrichment in each ChIP sample represented 1 biological replicate (3 replicates total). Two PpMYB5 promoter regions were assessed for enrichment. Primers are listed in Table S1.

## Supplementary Material

Web_Material_uhaf300

## Data Availability

The original contributions presented in the study are included in Supplementary Materials.
