# Supplementary material for: A novel R2R3-MYB transcription factor PpMYB5 assisting Ppbbx24-del positively regulates anthocyanin biosynthesis in ‘Red Zaosu’ pear
Source: Hortic Res. 2025 Oct 29;13(2):uhaf300. doi: 10.1093/hr/uhaf300 (PMC12933668; doi:10.1093/hr/uhaf300)
Supplement: Web_Material_uhaf300 [file web_material_uhaf300.zip › Figure S8.pdf]

```
PpMYB5        MRNPSPSSKAAAAAASAKMQTTITASSSSSKAAGVAGGTKTPCCAKVGLKRGPWTPEEDE   60
Pdr11g025100  MRNPSPSSKAAAAAASAKMQTTITASSSSSKAAGVAGGTKTPCCAKVGLKRGPWTPEEDE   60
PbMYB5        MRNPPSSSKATAAAS.....ATMTTASTSSSKAGIAGGSKTPCCVKVGLKRGPWTPEEDE   55
Pdr3g017680   MRNPPSSSKATAAAS.....ATMTTASPSSSKAGIAGGSKTPCCVKVGLKRGPWTPEEDE   55
Consensus     mrnp  sska aaa       t t  s ss  ag agg ktpcc kvglkrgpwtpeede

PpMYB5        LLANYIKKEGEGRWTRLPKRAGLLRCGKSCRLRWMNYLRPSVKRGQIAPDEEDLILRLHR  120
Pdr11g025100  LLANYIKKEGEGRWTRLPKRAGLLRCGKSCRLRWMNYLRPSVKRGQIAPDEEDLILRLHR  120
PbMYB5        LLANYIKKEGEGRWTRLPKQAGLLRCGKSCRLRWMNYLRPSVKRGQIAPDEEDLILRLHR  115
Pdr3g017680   LLANYIKKEGEGRWTRLPKQAGLLRCGKSCRLRWMNYLRPSVKRGQIAPDEEDLILRLHR  115
Consensus     llanyikkegegrwtrlpk agllrcgkscrlrwmnylrpsvkrgqiapdeedlilrlhr

PpMYB5        LLGNRWSLIAGRIPGRTDNEIKNYWNTHLSKKLINQGIDPRTHKPLNPDHHSAADDADLD  180
Pdr11g025100  LLGNRWSLIAGRIPGRTDNEIKNYWNTHLSKKLINQGIDPRTHKPLNPDHHSAADDADLD  180
PbMYB5        LLGNRWSLIAGRIPGRTDNEIKNYWNTHLSKKLISQGIDPRTHKPLNPDHHSAAADADVD  175
Pdr3g017680   LLGNRWSLIAGRIPGRTDNEIKNYWNTHLSKKLISQGIDPRTHKPLNPDHHSAAADADVD  175
Consensus     llgnrwsliagripgrtdneiknywnthlskkli qgidprthkplnpdhhsaa dad d

PpMYB5        NTNKSTAVASSSKANDRFSNPNPSPP.SDRLVHKEGDPNNSRNGGNIAIDDHDQGTIVHG  239
Pdr11g025100  NTNKSTAVASSSKANDRFSNPNPSPP.SDRLVHKEGDPNNSRNGGNIAIDDHDQGTIVHG  239
PbMYB5        NTNKLVAAAASFKANTRFSNPNPSPPPSDRLVHQGADPSINGNDGNIAID.HDLGTIVHS  234
Pdr3g017680   NTNKSVAAAVSFKANTRFSNPNPSPPPSDRLVHQGADPSIN...................  216
Consensus     ntnk  a a s kan rfsnpnpspp sdrlvh   dp

PpMYB5        YANMITSINNPDASSSATATGTLSLRSNN.SHGGVLLGGGGNEEDDDINCCADDVFSSFL  298
Pdr11g025100  YANMITSINNPDASSSATATGTLSLRSNN.SHGGVLLGGGGNEEDDDINCCADDVFSSFL  298
PbMYB5        CANLITSINNPDASSSAAAMGTSSLRTNNNSQAGVLLGGGGNEEDEDINCCADDVFSSFL  294
Pdr3g017680   ..................AMGTLSLRTNNNSHAGVLLGGGGNEEDEDINCCADDVFSSFL  258
Consensus                       a gt slr nn s  gvllggggneed dinccaddvfssfl

PpMYB5        NSLINEDPFHGQHQLQQ.VLQNGNVSAHAAAAGSENLPLITMTGASTTAPSTFGWESAVL  357
Pdr11g025100  NSLINEDPFHGQHQLQQ.VLQNGNVSAHAAAAGSENLPLITMTGASTTAPSTFGWESAVL  357
PbMYB5        NSLINEDPFAVQHQLQQQVLHNGNVSTHAAGAGSDHVPLISMTSASTMVPSTFGWDSAVL  354
Pdr3g017680   NSLINEDPFAVQHQLQQQVLHNGNVSTHAAGAGSDHVPLISMTSASTMTPSTFGWDSAVL  318
Consensus     nslinedpf  qhqlqq vl ngnvs haa ags   pli mt ast  pstfgw savl

PpMYB5        MSSAFIHNDHQRVNDPTE                                            375
Pdr11g025100  MSSAFIHNDHQRVNDPTE                                            375
PbMYB5        MSSAFIQNDHQRVTDQTE                                            372
Pdr3g017680   MSSAFIQNDHQRVTDQTE                                            336
Consensus     mssafi ndhqrv d te
```
