# Supplementary material for: A novel R2R3-MYB transcription factor PpMYB5 assisting Ppbbx24-del positively regulates anthocyanin biosynthesis in ‘Red Zaosu’ pear
Source: Hortic Res. 2025 Oct 29;13(2):uhaf300. doi: 10.1093/hr/uhaf300 (PMC12933668; doi:10.1093/hr/uhaf300)
Supplement: Web_Material_uhaf300 [file web_material_uhaf300.zip › Figure S9.pdf]

```
PpMYB5    ATGAGGAACCCATCGCCTTCGTCGAAAGCAGCAGCAGCAGCAGCAAGTGCTAAGATGCAAACGACGATAACAGCGTCGTC     80
PbMYB5    ATGAGGAACCCCCCGTCGTCGTCGAAAGCAACAGCAGCAG...CAAGTGCTA.........CGATGACAACAGCATCGAC     68
Consensus atgaggaaccc  cg c tcgtcgaaagca cagcagcag   caagtgcta         cga ga aacagc tcg c

PpMYB5    CTCGTCGAGCAAGGCGGCTGGGGTTGCTGGAGGGACCAAGACGCCGTGTTGCGCAAAGGTGGGTTTGAAGAGAGGGCCGT    160
PbMYB5    GTCGTCGAGTAAGGCGG...GGATTGCCGGAGGGAGTAAGACGCCGTGTTGTGTAAAGGTGGGTTTGAAGAGGGGGCCGT    145
Consensus  tcgtcgag aaggcgg   gg ttgc ggaggga  aagacgccgtgttg g aaaggtgggtttgaagag gggccgt

PpMYB5    GGACTCCCGAAGAGGACGAGCTGCTGGCAAATTACATCAAGAAAGAAGGGGAGGGACGGTGGCGGACCCTTCCCAAGCGG    240
PbMYB5    GGACTCCTGAAGAGGACGAGCTGCTGGCCAATTACATCAAGAAAGAAGGTGAAGGACGGTGGCGGACCCTCCCCAAGCAG    225
Consensus ggactcc gaagaggacgagctgctggc aattacatcaagaaagaagg ga ggacggtggcggaccct cccaagc g

PpMYB5    GCTGGGTTGCTCCGCTGCGGTAAGAGCTGCCGCCTCCGCTGGATGAACTATCTCCGCCCCTTCCGTAAAGCGCGGCCAGAT    320
PbMYB5    GCTGGGTTGCTCCGCTGCGGAAAAAGCTGTCGCCTCCGCTGGATGAACTACCTCCGCCCCTTCCGTTAAGCGCGGCCAGAT    305
Consensus gctgggttgctccgctgcgg aa agctg cgcctccgctggatgaacta ctccgccccttccgt aagcgcggccagat

PpMYB5    CGCCCCCGATGAAGAAGATCTCATCCTTCGCCTCCATCGCCTTCTGGGCAATCGGTGGTCTTTGATAGCTGGGAGGATTC    400
PbMYB5    CGCCCCCGATGAAGAAGATCTCATTCTCCGCCTCCATCGCCTCCTCGGCAATCGGTGGTCTTTGATAGCTGGGAGGATTC    385
Consensus cgcccccgatgaagaagatctcat ct cgcctccatcgcct ct ggcaatcggtggtctttgatagctgggaggattc

PpMYB5    CAGGCCGTACGGACAATGAGATAAAGAACTACTGGAACACACACCTGAGCAAGAAGCTGATAAACCAAGGCATAGATCCC    480
PbMYB5    CAGGTCGTACGGACAATGAGATAAAGAACTACTGGAACACACACCTGAGCAAGAAGCTGATAAGCCAAGGCATAGATCCC    465
Consensus cagg cgtacggacaatgagataaagaactactggaacacacacctgagcaagaagctgataa ccaaggcatagatccc

PpMYB5    AGAACCCACAAGCCTCTCAATCCAGATCATCACTCTGCTGCTGATGATGCTGACCTGGACAACACAAACAAATCAACTGC    560
PbMYB5    AGAACCCACAAGCCTCTCAATCCAGATCATCACTCTGCTGCTGCCGATGCTGATGTGGACAACACAAATAAATTAGTTGC    545
Consensus agaacccacaagcctctcaatccagatcatcactctgctgctg  gatgctga  tggacaacacaaa aaat a  tgc

PpMYB5    TGTTGCTTCTTCTTCCAAAGCCAATGATCGGTTCTCAAACCCTAACCCTAGTCCCCCT...TCTGATCGTCTTGTCCATA    637
PbMYB5    TGCTGCTGCTTCTTTCAAGGCTAATACCCGGTTCTCAAACCCTAATCCTAGTCCTCCTCCTTCTGATCGTCTTGTCCATC    625
Consensus tg tgct cttctt caa gc aat   cggttctcaaaccctaa cctagtcc cct   tctgatcgtcttgtccat

PpMYB5    AAGAAGGGGATCCAAATAACAGCCGTAATGGTGGAAACATCGCAATTGATGATCATGATCAGGGCACTATAGTCCATGGC    717
PbMYB5    AAGGAGCGGATCCCAGTATCAACGGTAATGATGGAAACATCGCAATTGAT...CATGATCTGGGTACTATAGTCCATAGC    702
Consensus aag ag ggatcc a ta ca c gtaatg tggaaacatcgcaattgat   catgatc ggg actatagtccat gc

PpMYB5    TATGCAAATATGATCACGTCCATCAACAATCCCGATGCTTCTTCTTCGGCCACGGCAACGGGTACTTTGAGTTTGAGGA.    796
PbMYB5    TGTGCAAACTTGATCACGTCCATTAACAATCCCGATGCTTCTTCTTCGGCCGCAGCAATGGGCACTTCGAGTTTAAGGAC    782
Consensus t tgcaaa  tgatcacgtccat aacaatcccgatgcttcttcttcggcc c gcaa ggg actt gagttt agga

PpMYB5    ..GCAACAACAGCCACGGTGGAGTACTACTTGGGGGAGGAGGAAATGAAGAGGACGACGACATCAACTGTTGTGCGGACG    874
PbMYB5    CAACAACAACAGCCAGGCTGGAGTACTACTTGGGGGAGGAGGAAATGAAGAGGACGAGGACATCAACTGTTGTGCGGACG    862
Consensus    caacaacagcca g tggagtactacttgggggaggaggaaatgaagaggacga gacatcaactgttgtgcggacg

PpMYB5    ACGTCTTCTCTTCGTTTCTGAATTCGTTGATCAATGAGGATCCATTTCATGGACAACACCAATTGCAACAA...GTACTG    951
PbMYB5    ACGTCTTCTCTTCGTTTCTGAATTCGTTGATCAACGAGGATCCATTTGCTGTACAACACCAATTGCAACAACAGGTACTG    942
Consensus acgtcttctcttcgtttctgaattcgttgatcaa gaggatccattt  tg acaacaccaattgcaacaa   gtactg

PpMYB5    CAGAATGGGAATGTGAGTGCACACGCAGCTGCTGCTGGTTCCGAGAACCTTCCTTTGATTACTATGACTGGTGCTAGTAC   1031
PbMYB5    CACAATGGGAATGTTAGTACACACGCAGCTGGTGCTGGTTCCGACCACGTTCCTTTGATTTCTATGACTAGTGCTAGTAC   1022
Consensus ca aatgggaatgt agt cacacgcagctg tgctggttccga  ac ttcctttgatt ctatgact gtgctagtac

PpMYB5    TACGGCGCCATCAACATTTGGCTGGGAGTCTGCTGTGCTCATGTCTTCTGCTTTCATCCATAATGATCGCCAAAGGGTTA   1111
PbMYB5    TATGGTGCCGTCAACATTTGGCTGGGACTCTGCTGTGCTCATGTCTTCTGCTTTCATCCAAAATGATCACCAGAGGGTTA   1102
Consensus ta gg gcc tcaacatttggctggga tctgctgtgctcatgtcttctgctttcatcca aatgatc cca agggtta

PpMYB5    ATGATCCAACGGAGTAG..                                                                1128
PbMYB5    CTGATCAAACGGAGCAGTA                                                                1121
Consensus  tgatc aacggag ag
```
