# Supplementary figures and images for: A novel R2R3-MYB transcription factor PpMYB5 assisting Ppbbx24-del positively regulates anthocyanin biosynthesis in ‘Red Zaosu’ pear
Source: Hortic Res. 2025 Oct 29;13(2):uhaf300. doi: 10.1093/hr/uhaf300 (PMC12933668; doi:10.1093/hr/uhaf300)

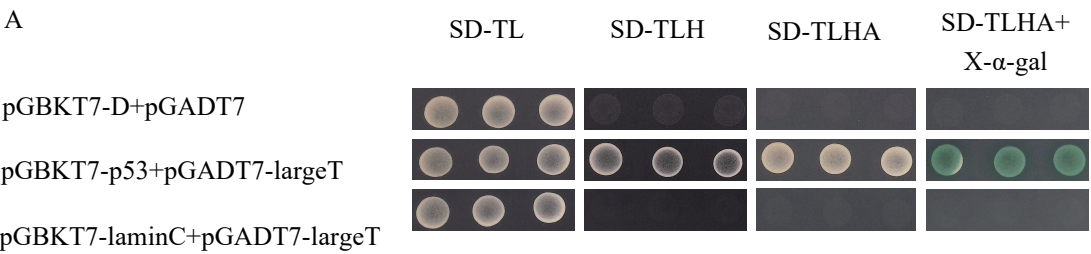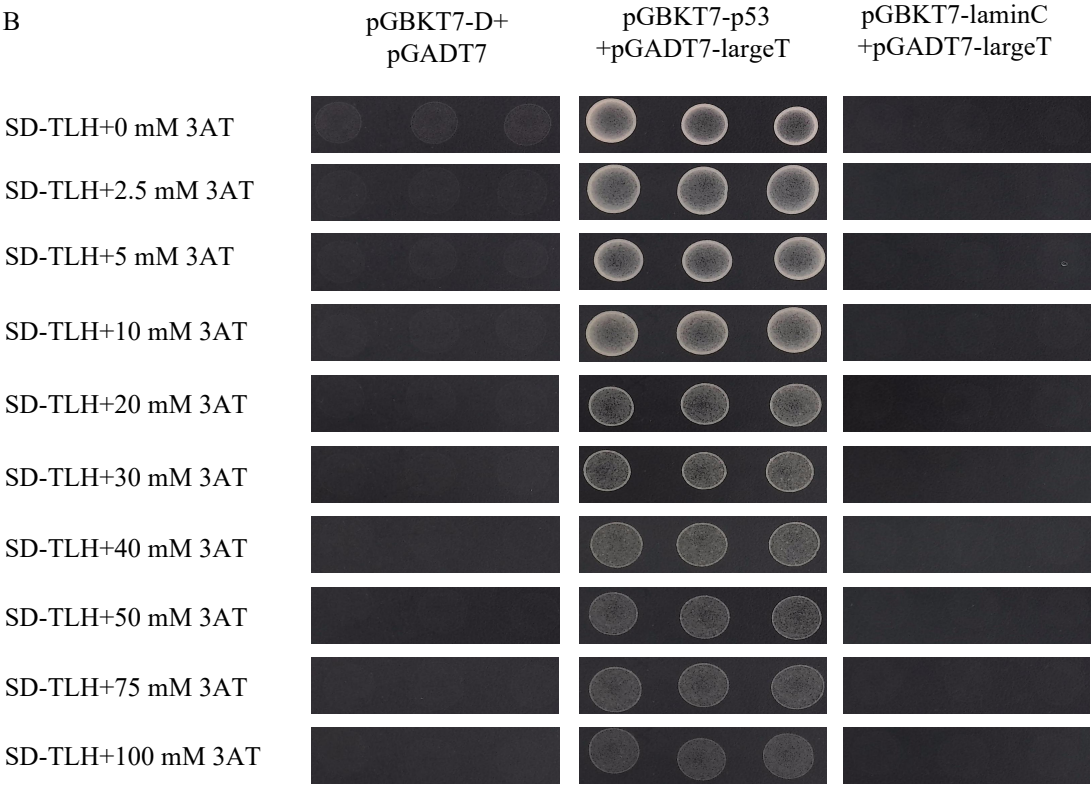

Supplement: Web_Material_uhaf300 [file web_material_uhaf300.zip › Figure S1.pdf]

SD-TL

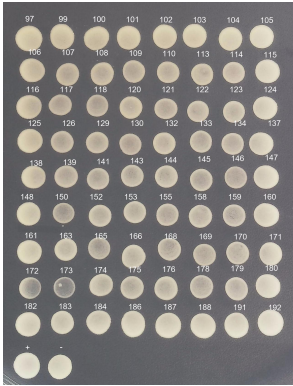

SD-TLH+2.5 mM 3AT

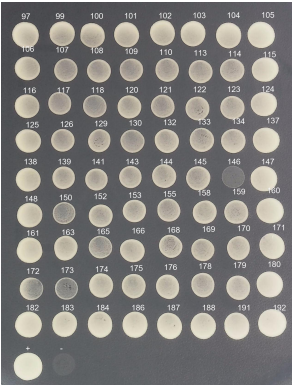

SD-TLH+2.5 mM 3AT +X- $\alpha$ -gal

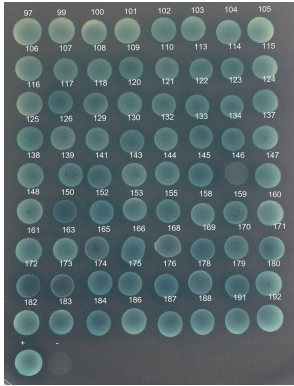

SD-TLHA+2.5 mM 3AT

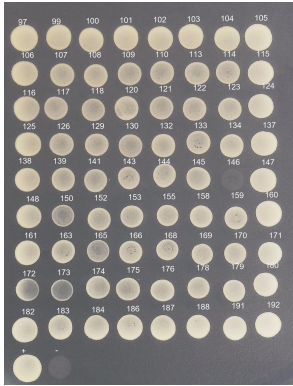

SD-TLHA+2.5 mM 3AT +X- $\alpha$ -gal

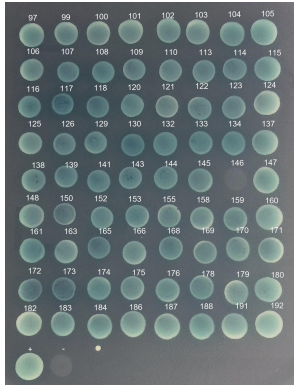

Supplement: Web_Material_uhaf300 [file web_material_uhaf300.zip › Figure S2.pdf]

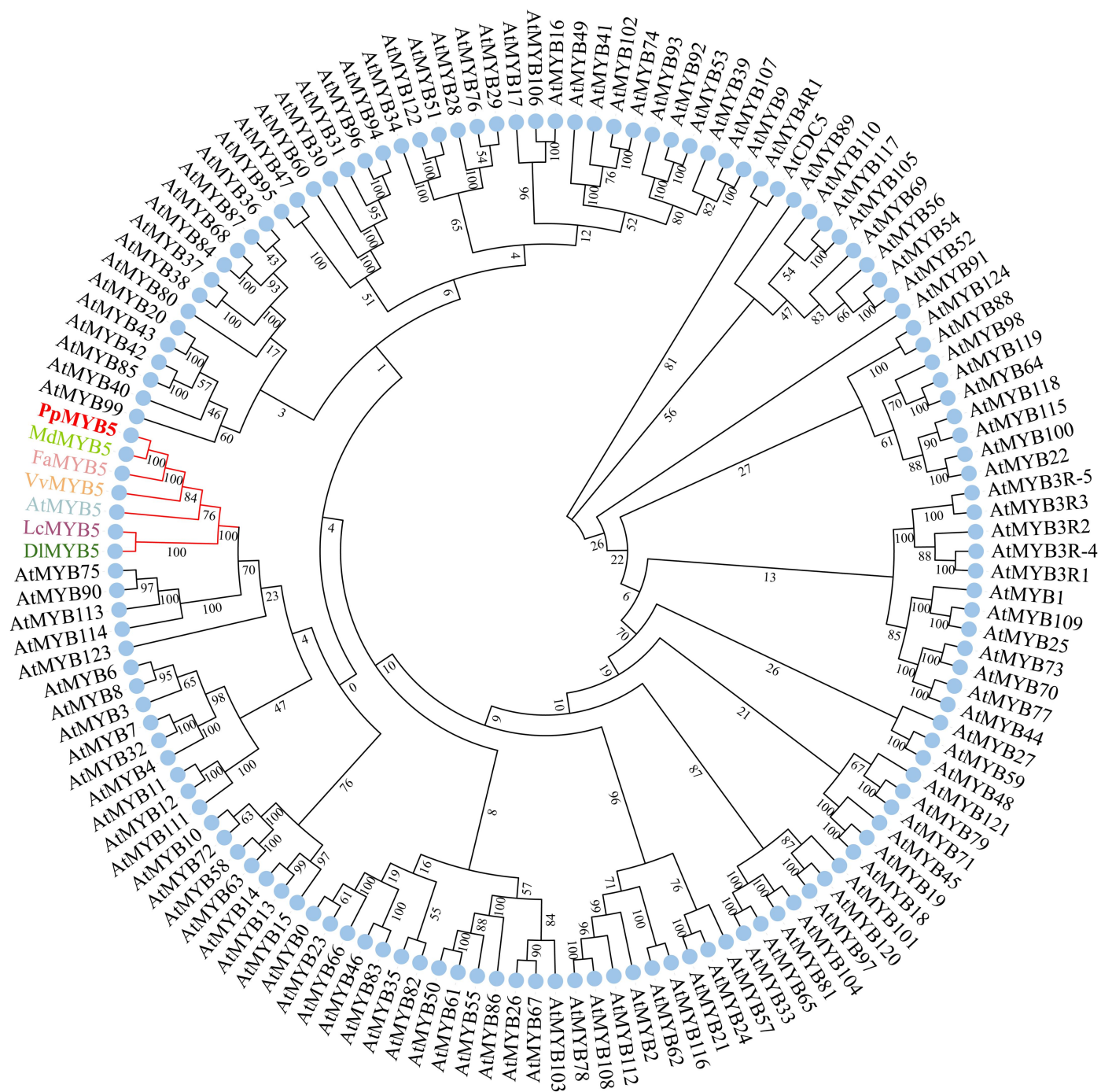

Supplement: Web_Material_uhaf300 [file web_material_uhaf300.zip › Figure S3.pdf]

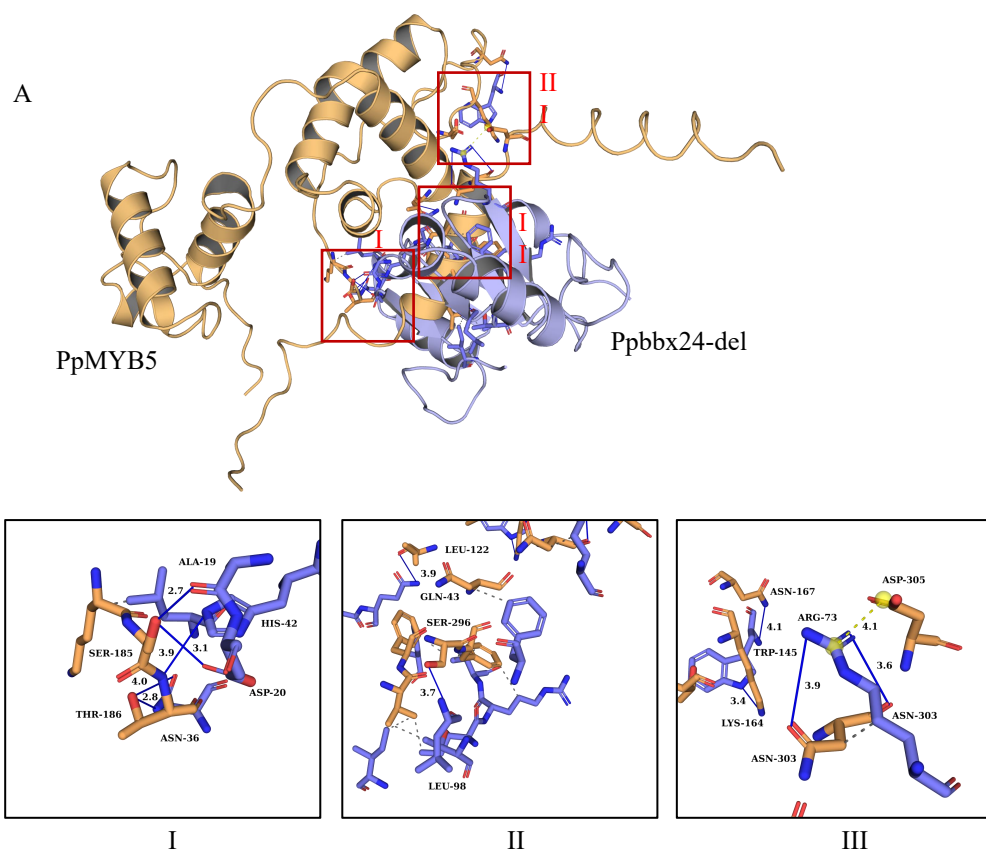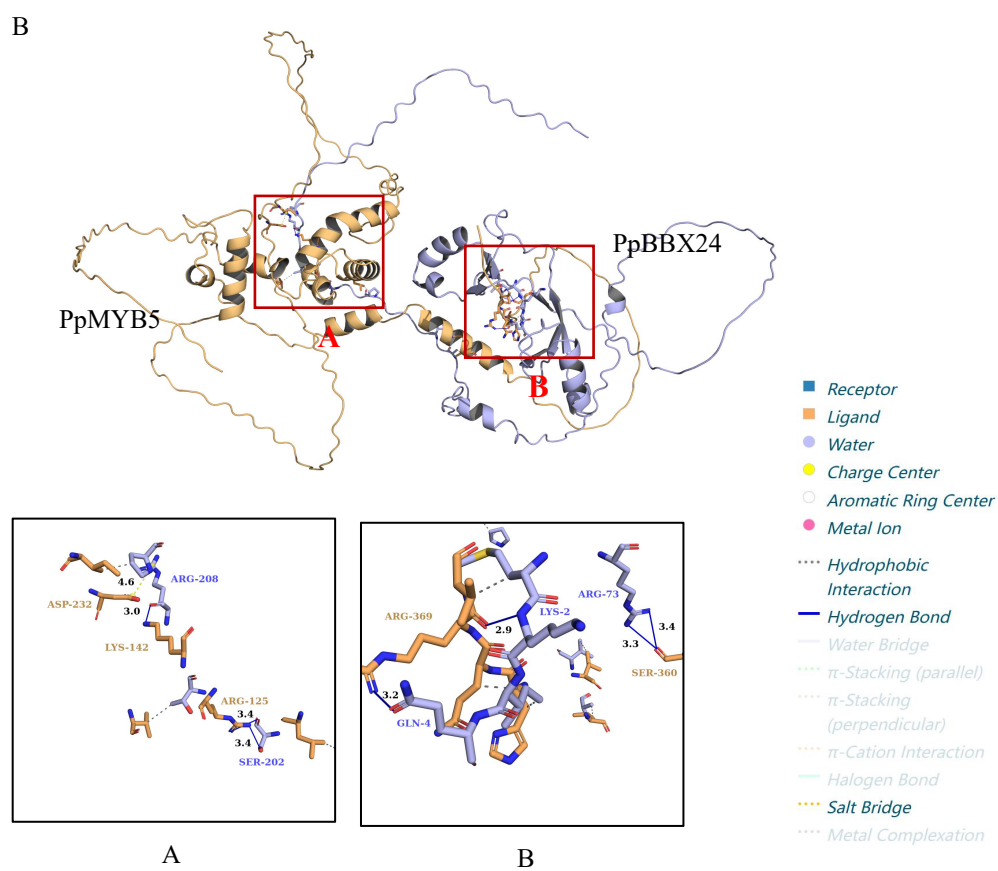

Supplement: Web_Material_uhaf300 [file web_material_uhaf300.zip › Figure S4.pdf]

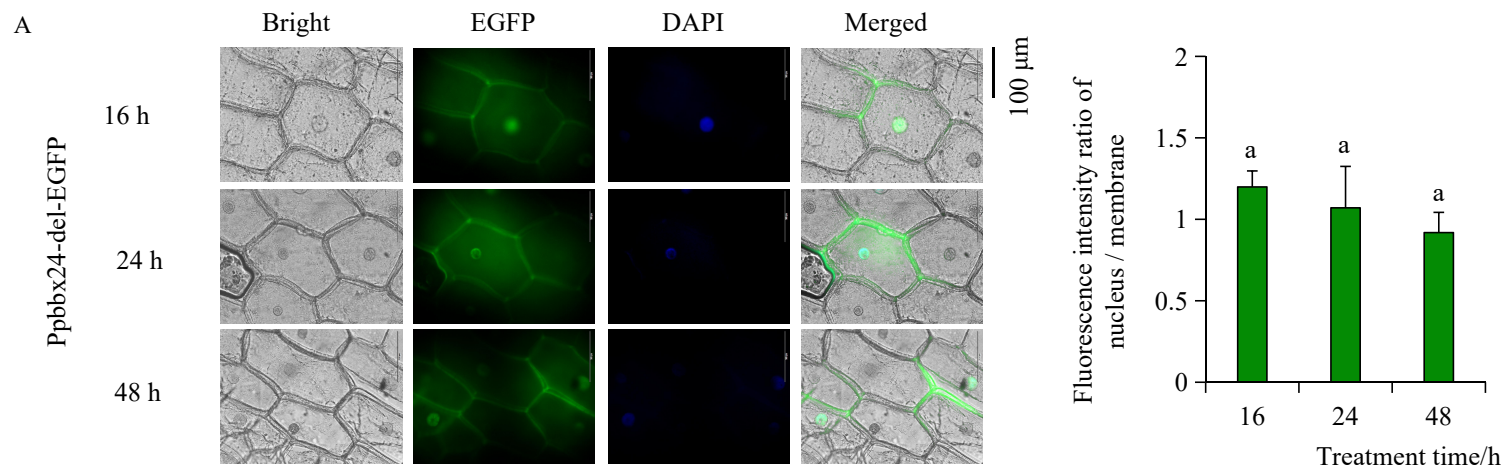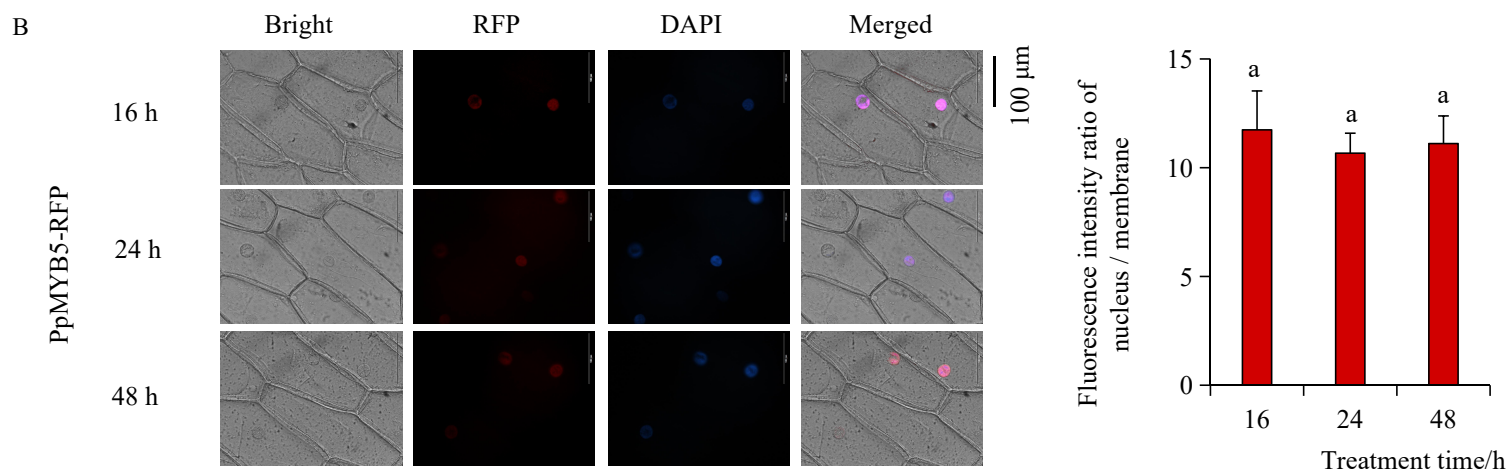

Supplement: Web_Material_uhaf300 [file web_material_uhaf300.zip › Figure S5.pdf]

A

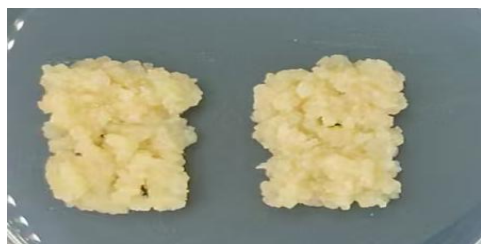

OE-PpMYB5

WT

B

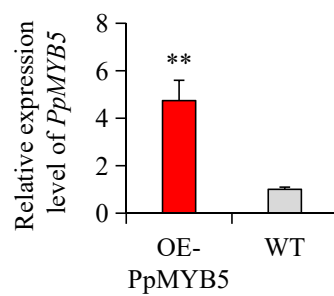

C

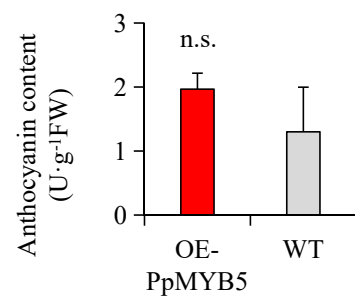

D

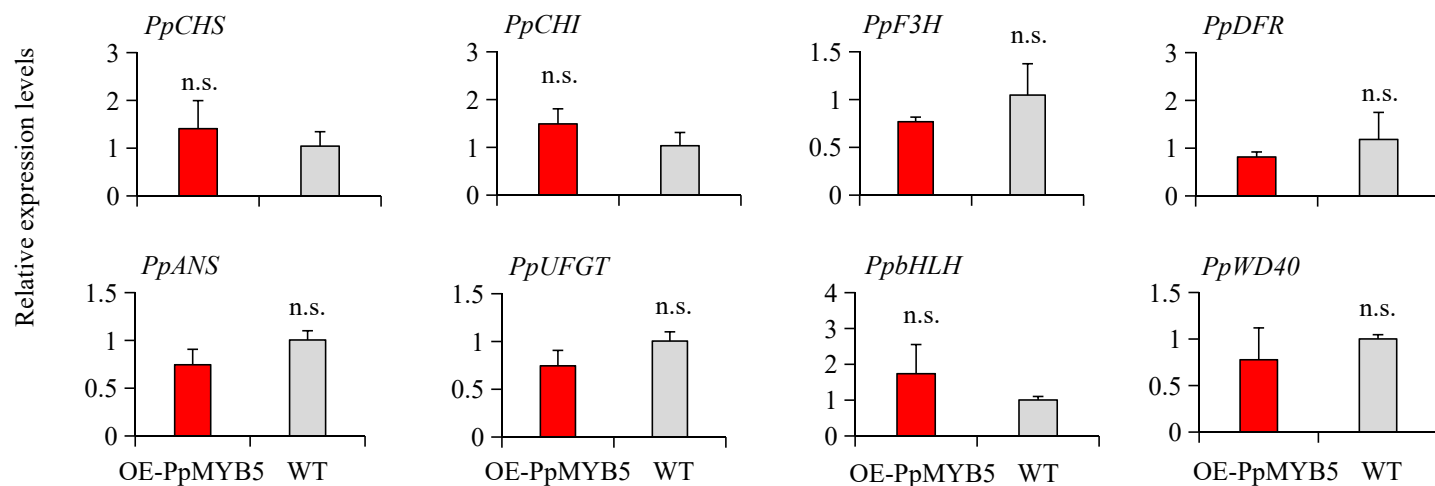

Supplement: Web_Material_uhaf300 [file web_material_uhaf300.zip › Figure S6.pdf]

PpMYB5

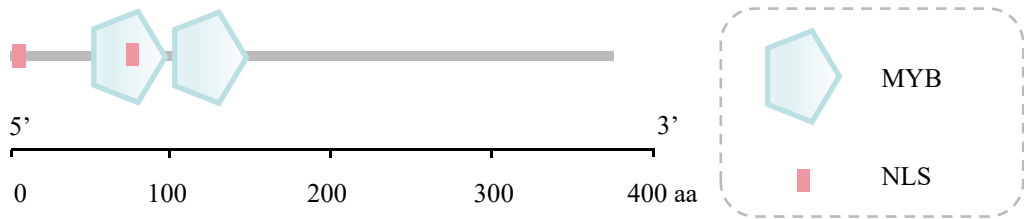

Supplement: Web_Material_uhaf300 [file web_material_uhaf300.zip › Figure S7.pdf]
